# Arthroscopic Refixation of the Medial Meniscal Posterior Root With an Intramedullary Titanium Screw

**DOI:** 10.1016/j.eats.2025.103577

**Published:** 2025-05-08

**Authors:** Nikita Vadimovich Vasilchenko, Ivan Ivanovich Radysh, Aleksandr Aleksandrovich Vetoshkin, Sergey Sergeevich Gusev, Ksenia Dmitrievna Mikhaylova

**Affiliations:** a Department of Traumatology and Rehabilitation, Federal State Budgetary Institution Volynskaya Clinical Hospital No. 1, Office of the President of the Russian Federation, Moscow, Russia; b Federal State Budgetary Educational Institution of Further Professional Education, Russian Medical Academy of Continuous Professional Education, Ministry of Healthcare of the Russian Federation, Moscow, Russia; c Traumatology and Orthopaedics Department, European Clinic of Sports Traumatology and Orthopedics, Moscow, Russia; d Traumatology and Orthopedics Department, Federal State Public Enterprise Nikiforov’s All-Russian Center for Emergency and Radiation Medicine of the Emergencies Ministry of Russia, St. Petersburg, Russia

## Abstract

Arthroscopic repair of medial meniscal posterior root tears addresses a complex area of modern traumatology. Meniscal posterior root tear presents challenges owing to its potential post-traumatic consequences, including functional deficiency, as well as increased risk of degenerative changes and osteoarthritis, which significantly deteriorate patients' quality of life. The considerable interest from both clinicians and researchers stems from the serious implications of such injuries, the diagnostic challenges—particularly in the early stages—and the inconsistent clinical outcomes associated with various treatment modalities. Despite the availability of diverse surgical approaches and substantial clinical experience, the prevalence of unsatisfactory results indicates that meniscal posterior root tear remains a complex issue yet to be fully resolved. Moreover, a consensus on the optimal management strategy for this injury has not been established. Consequently, we present an effective, safe, and reproducible technique for arthroscopic intramedullary refixation of the medial meniscal posterior root with a titanium screw, using only standard arthroscopic tools to minimize additional costs.

In addition to its primary role as a shock absorber that mitigates axial loads, the medial meniscus serves as a secondary stabilizer of the knee joint.^1^ The medial meniscal posterior root is critical for stabilizing the knee during anterior tibial translation and rotational loads.^2^ Ruptures of this root account for approximately 20% of all meniscal tears,^3^ with significantly higher prevalence rates observed in specific populations, particularly individuals older than 50 years, those with a high body mass index, and those with low levels of physical activity.^4^ Biomechanical studies have shown that damage to the posterior root is similar to a total meniscectomy, leading to accelerated degenerative changes in articular cartilage and resulting in severe osteoarthritis of the knee.^5^

Conservative treatment is generally ineffective in restoring the stabilizing function of the meniscal root; however, it may be considered for elderly or obese patients and in cases in which surgical intervention is contraindicated.^6^ Such conservative approach typically involves symptomatic treatment with nonsteroidal anti-inflammatory drugs, lifestyle modifications, physiotherapy, and strategies to reduce the load on the knee joint.^7^

The crucial biomechanical role of the posterior root necessitates early surgical intervention, particularly in young and active patients with minimal articular cartilage damage. Currently, the transtibial repair technique is regarded as the gold standard for the surgical management of this condition because it provides anatomic fixation of the damaged segment while restoring knee stability and kinematics.^8^ This technique allows for precise manipulation in the selection of suture passage points; a channel is created in the anterior projection of the proximal tibia to the anatomic attachment of the medial meniscal posterior root, through which fixation threads are passed and secured with extracortical devices.

Clinical outcomes have consistently shown significant improvements in function, activity levels, and pain intensity.^9^ Nonetheless, the challenge of achieving complete restoration of the native biomechanics of the knee remains unresolved.^10^ This technical note aims to provide a detailed description of arthroscopic intramedullary refixation of the medial meniscal posterior root using a titanium screw in the tibial canal, using standard arthroscopic approaches. A summary of the surgical technique is presented in Video 1.

## Surgical Technique

**Fig 1:**
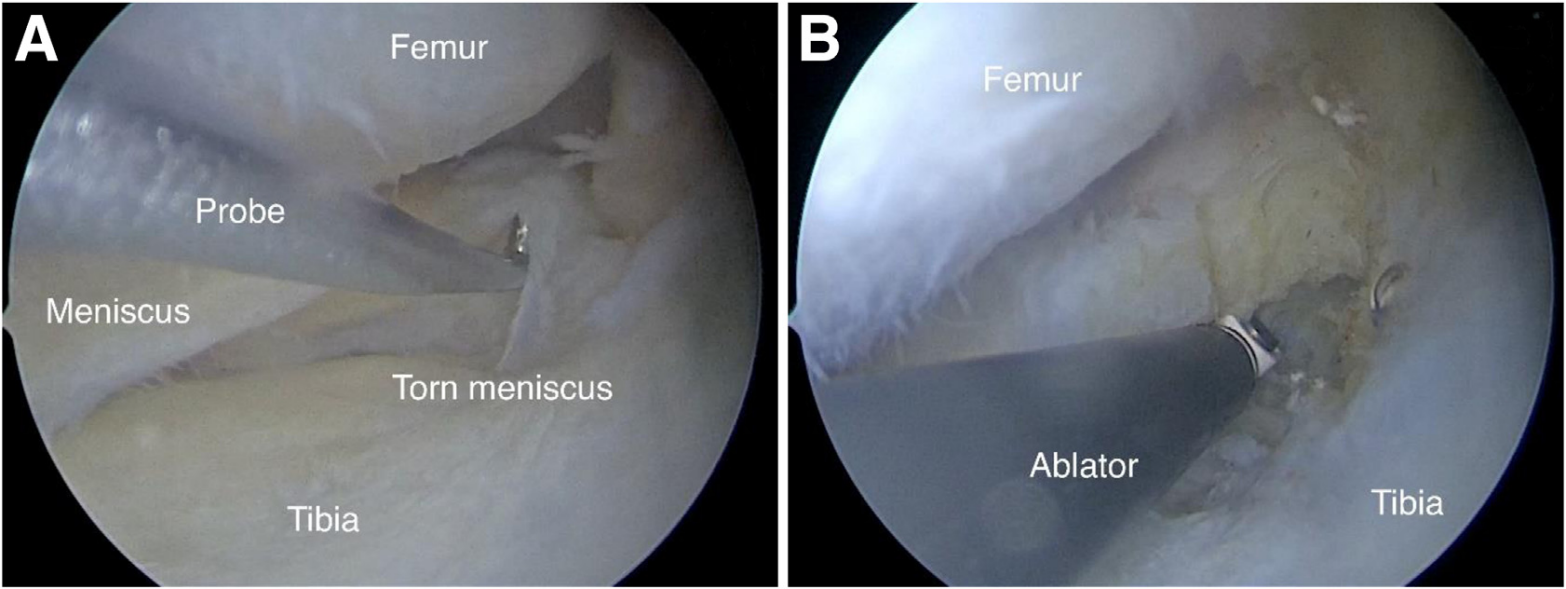
The patient is in the supine position. (A) Arthroscopic view from anterolateral portal to identify damage to medial meniscal posterior root of left knee using arthroscopic hook. (B) Soft-tissue release and release of damaged fragment of posterior root via arthroscopic ablation.

Standard diagnostic arthroscopy is performed through the anterolateral portal of the knee joint. Under arthroscopic visualization, a 25-gauge needle is applied to partially release the medial collateral ligament at the site of the tibial fixation. An anteromedial approach is subsequently established, using a 25-gauge needle for precise guidance by loading an arthroscopic hook into the knee joint cavity. This maneuver aims to assess the extent of damage to the medial meniscal posterior root (Fig 1A). Soft-tissue release and release of the damaged proximal fragment of the posterior root are accomplished via ablation, which enhances visualization and prepares the bone bed for the refixation of the meniscal root (Fig 1B).

**Fig 2:**
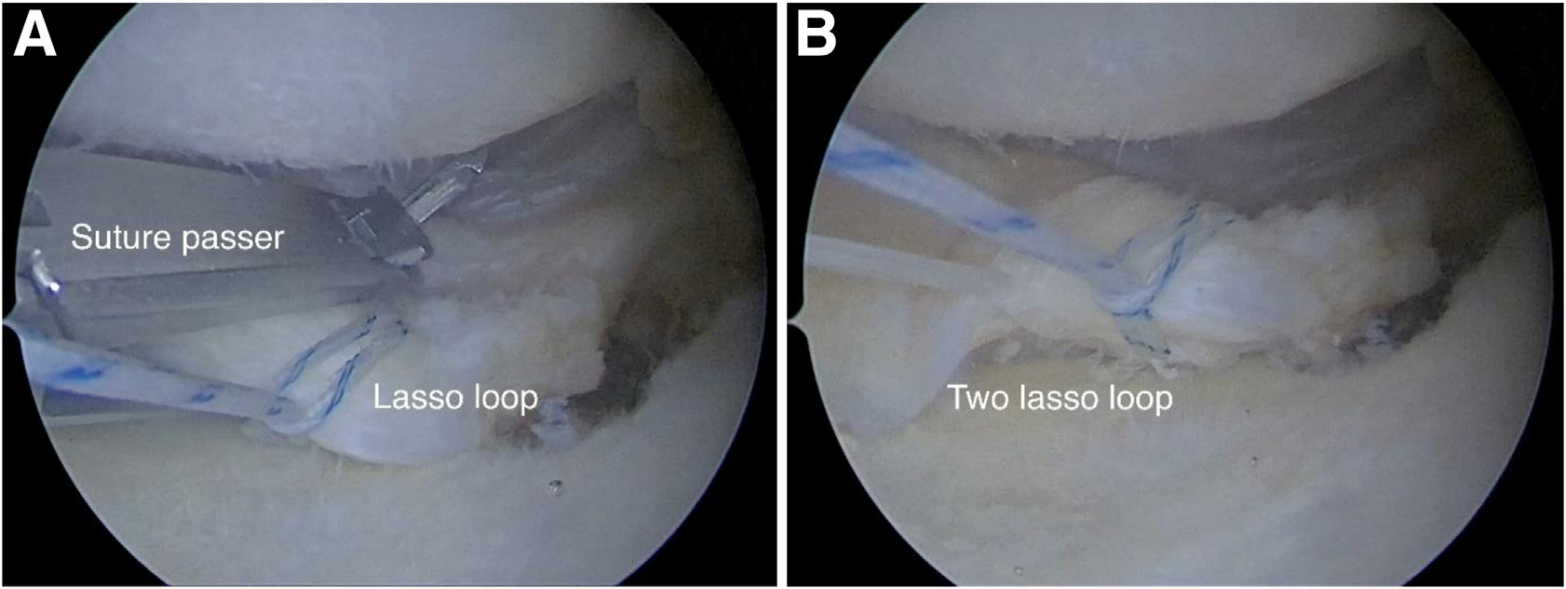
Arthroscopic view from anterolateral portal of left knee with patient in supine position. (A) Suturing of medial meniscal posterior root with semi-automatic suture passer (Zimmer Quattro Suture Passer). (B) Formation of 2 lasso-loop knots.

**Fig 3:**
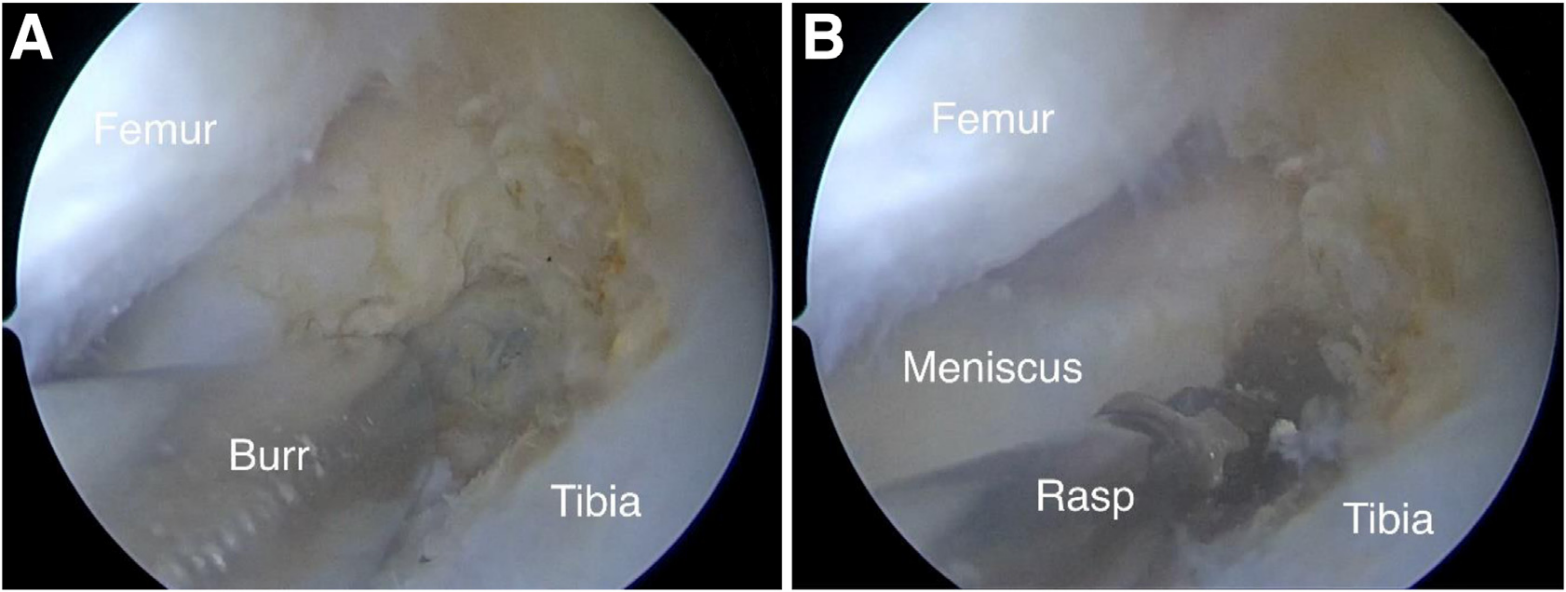
Arthroscopic view from anterolateral portal of left knee with patient in supine position. Preparation of bone bed: decortication using burr (A) and rasp (B).

Decortication is performed using both a burr and an arthroscopic rasp (Fig 2). During the suturing of the distal portion of the torn posterior root, a semi-automatic suture passer (Zimmer Quattro Suture Passer; Zimmer Biomet Holdings, Warsaw, IN)—specifically designed for rotator cuff repair—is used (Fig 3A). A lasso-loop knot is formed to enhance fixation reliability. An additional suture is placed to create another lasso-loop knot using the same semi-automatic device (Fig 3B).

**Fig 4:**
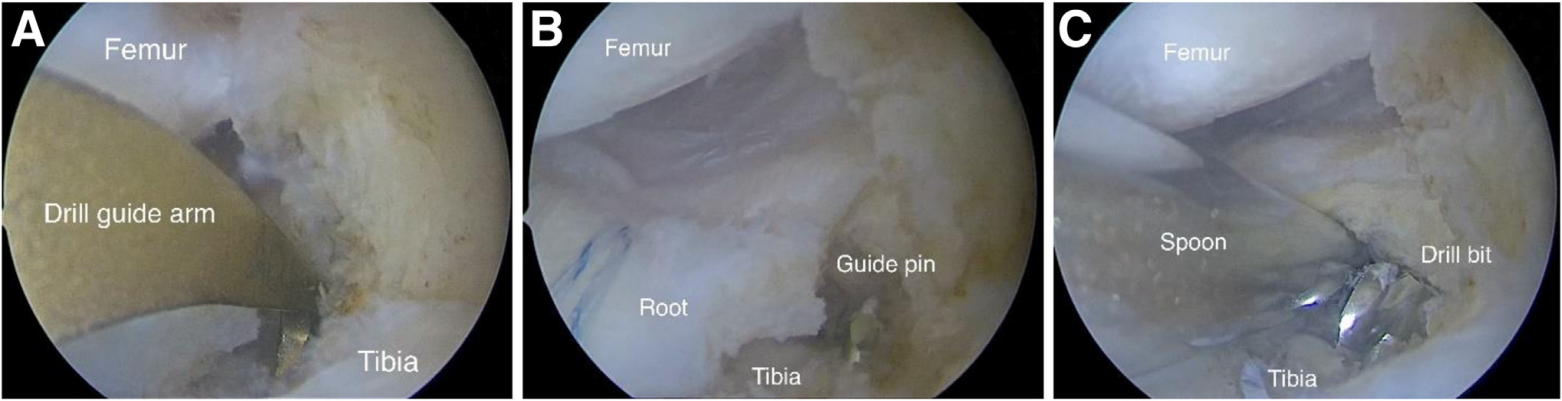
Arthroscopic view from anterolateral portal of left knee with patient in supine position. (A) Installation of guide (Acufex) in anatomic fixation area of medial meniscal posterior root. (B) Insertion of guide pin. (C) Formation of transtibial canal 6 mm in diameter.

A guide (Acufex; Smith & Nephew, Vantaa, Finland) is positioned in the anatomic area of the posterior root attachment site; in this instance, a standard tibial guide arm used for anterior cruciate ligament reconstruction is used (Fig 4A). A guide pin is inserted through the guide into the posterior root fixation area (Fig 4B). A 6-mm transtibial canal is then created along the guide pin to establish a bone bed for the refixed meniscal posterior root (Fig 4C).

**Fig 5:**
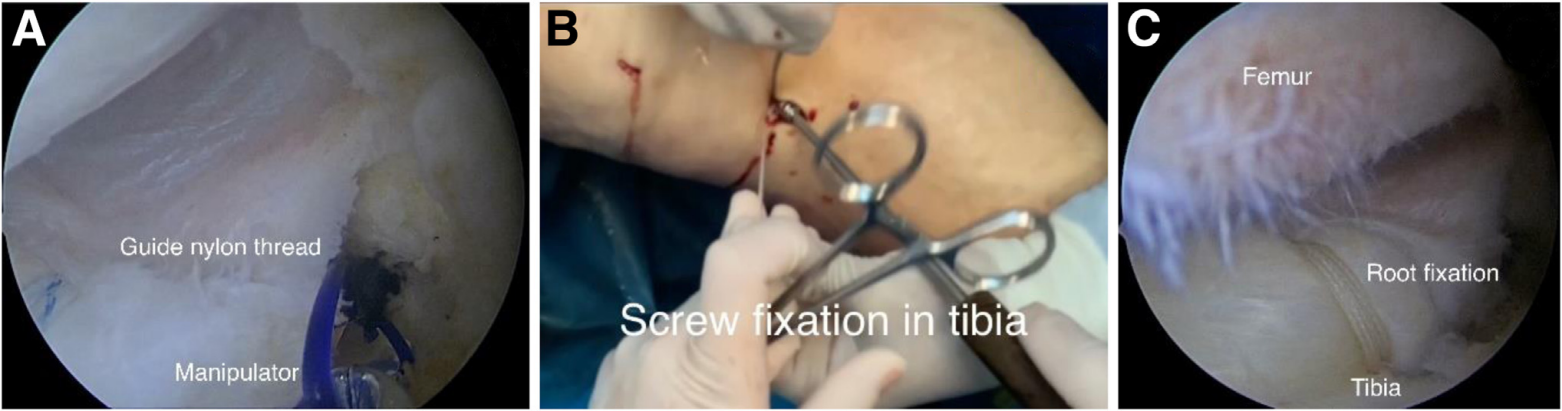
Arthroscopic view from anterolateral portal of left knee with patient in supine position. (A) The PDS guiding thread (Ethicon, Johnson&Johnson) passed through the drill brings out the fixation threads of the meniscal root to the anterior surface of the tibia. (B) Intramedullary fixation with a titanium screw (Arthrex, Naples, FL) is performed along the nitinol guide. (C) Final evaluation to confirm that anatomic firm fixation has been achieved.

Next, a PDS thread (Ethicon, Johnson&Johnson, Raritan, NJ) is passed through the drill and accessed via the medial arthroscopic portal. By use of the PDS thread, the sutures securing the posterior root are pulled in a transtibial direction and brought out through the anterior tibial surface (Fig 5A). A nitinol guidewire is inserted into the tibial canal. With the knee in 30° of flexion, intramedullary fixation with a titanium screw measuring 8 mm in diameter and 25 mm in length is performed along the nitinol guide, ensuring maximum tension is applied to the fixation threads (Fig 5B). The final stage involves an arthroscopic evaluation of the posterior root refixation and its stability (Fig 5C).

## Discussion

In the treatment of medial meniscal posterior root tears, surgical reconstruction is the preferred method for restoring the meniscus's shock-absorbing and stabilizing functions. The technique described for posterior root reconstruction offers several advantages over current methods; however, it is essential to acknowledge some limitations of this technique, as shown in Table 1. Pearls and pitfalls are presented in Table 2.

**Table 1:** Advantages and Disadvantages of Arthroscopic Refixation of Medial Meniscal Posterior Root With Intramedullary Titanium Screw

Advantages
The likelihood of healing is enhanced by the creation of a 6-mm-diameter bone tunnel at the anatomic attachment site, which increases the contact area of the meniscus-to-bone interface.
Stable and firm fixation is achieved through the use of lasso loop–type knots, which secure the distal portion of the meniscus with a larger contact area between the suture and meniscal surface.
This method reduces the risk of suture cutting during tension, which is particularly important in cases involving significant degenerative changes in the meniscus.
This surgical approach can be readily implemented in a standard arthroscopy without the need for specialized instruments; a semi-automatic rotator cuff suture passer is used for refixation, and a guide for anterior cruciate ligament reconstruction serves to form the transtibial canal.
This method is economically advantageous compared with extracortical fixation with anchors or cortical buttons because a titanium screw is used while ensuring comparable structural stability.
Disadvantages
The procedure carries a risk of damaging the neurovascular bundle in the popliteal region during canal formation.
There is a potential for migration of the titanium screw within the tibial canal.

**Table 2:** Pearls and Pitfalls of Arthroscopic Refixation of Medial Meniscal Posterior Root With Intramedullary Titanium Screw

Pearls
Partially release the medial collateral ligament to minimize the risk of cartilage injury, improve visualization of the anatomic attachment site, and facilitate visual control during transtibial tunnel formation.
Perform decortication with a burr or arthroscopic rasp to increase the meniscal contact surface area, thereby enhancing healing potential.
Pitfalls
Use a protector, such as an external tube from a burr attachment, when forming the transtibial canal to prevent injury to the neurovascular bundle.

Overall, the described technique for arthroscopic intramedullary refixation of the medial meniscal posterior root with a titanium screw appears promising, safe, and reproducible. It fosters favorable clinical outcomes through stable fixation and requires minimal training for practicing arthroscopists while avoiding additional expenses.

## Disclosures

All authors (N.V.V., I.I.R., A.A.V., S.S.G., K.D.M.) declare that they have no known competing financial interests or personal relationships that could have appeared to influence the work reported in this paper.

## Supplementary Data

Video 1 Technique for arthroscopic intramedullary refixation of medial meniscal posterior root with titanium screw, performed within tibial canal, through conventional arthroscopic portal on the left knee joint.
